# A pan-genomic approach to genome databases using maize as a model system

**DOI:** 10.1186/s12870-021-03173-5

**Published:** 2021-08-20

**Authors:** Margaret R. Woodhouse, Ethalinda K. Cannon, John L. Portwood, Lisa C. Harper, Jack M. Gardiner, Mary L. Schaeffer, Carson M. Andorf

**Affiliations:** 1 Corn Insects and Crop Genetics Research Unit, USDA-ARS, Ames, IA 50011 USA; 2 Division of Animal Sciences, University of Missouri, 65211 Columbia, MO USA; 3 Division of Plant Sciences, University of Missouri, 65211 Columbia, MO USA; 4 Department of Computer Science, Iowa State University, Ames, IA 50011 USA

**Keywords:** Databases, Genomes, Maize, Pan-genome, Nomenclature, Browsers, NAM founders

## Abstract

Research in the past decade has demonstrated that a single reference genome is not representative of a species’ diversity. MaizeGDB introduces a pan-genomic approach to hosting genomic data, leveraging the large number of diverse maize genomes and their associated datasets to quickly and efficiently connect genomes, gene models, expression, epigenome, sequence variation, structural variation, transposable elements, and diversity data across genomes so that researchers can easily track the structural and functional differences of a locus and its orthologs across maize. We believe our framework is unique and provides a template for any genomic database poised to host large-scale pan-genomic data.

**Supplementary Information:**

The online version contains supplementary material available at 10.1186/s12870-021-03173-5.

## Background

Zea mays ssp. mays (maize, corn) is a unique model organism as its broad importance as a food, feed, and fiber product has driven its domestication over thousands of years by the traditional breeding practices of indigenous people [1–3], followed by decades of directed breeding since the green revolution and the molecular era [4, 5]. Over the last 100 years, research on maize has also been instrumental in understanding plant biology, evolution, domestication, development, and genetics [6–10]. For over a decade, maize has been the world’s top production grain crop (http://faostat.fao.org/), primarily for livestock feed and more recently for biofuels [11].

Collection and sharing of maize research data, such as compiling gene lists, composite genetic maps, and breeding information, has been ongoing since the early twentieth century [9, 12]. Curated maize research data was formally moved into a database in 1991 (MaizeDB) [13]. The database expanded to include sequence data in 2003 [14] and genomic data in 2008, and by 2015, had evolved into the present Maize Genetics and Genomics Database (MaizeGDB – https://www.maizegdb.org) [15]. MaizeGDB is the maize community database, providing data curation and informatics resources to support maize genetics, genomics, and breeding research for maize scientists. MaizeGDB is also the maize research community’s hub, providing support, outreach, and training to facilitate collaboration and data sharing, and serves as the clearing house for maize genetic and genomic nomenclature.

By 2019, MaizeGDB hosted the genomes of six maize inbred lines and one teosinte [16]. Since then, MaizeGDB has brought in 39 additional reference-quality genomes, including important individual inbred lines (PH207 [17], Mo17 [18, 19], and W22 [20]), a set of European lines [21], a sweet corn [22], and the set of 26 high-quality PacBio genome assemblies of the Nested Associated Mapping (NAM) population founder lines [23]. The NAM founder lines represent a large swath of maize’s considerable diversity [24] and the resulting NAM populations have been used extensively by researchers to study maize flowering time [25], leaf architecture [26], disease resistance [27], and other important agronomic traits [28]. The sequencing, assembly, gene model annotation, RNA-seq expression data, structural variation, transposable element annotation, and methylome data of the NAM founder genomes were all performed using the same protocols in the same laboratories. Since comparisons between genome assemblies have been hampered by the difficulty of teasing out true biological differences from differences in assembly and annotation quality or techniques, the NAM founder genome assemblies and their associated data provide a unique opportunity to explore biologically relevant genome diversity within a single species. In hosting the NAM founder genomes and their data, MaizeGDB achieved in a single database update 26 new genome project/metadata pages; over 1 million new gene model pages; hundreds of new downloadable datasets; 134 additional BLAST targets; and 26 new JBrowse genome browsers with over 1,000 total tracks of data across the browsers. We use this massive gold-standard data set to develop new approaches to host and connect these genomes and their datasets to each other in a way that is useful and biologically meaningful to the maize community.

Research in the past decade has clearly demonstrated that a single reference genome is not truly representative of a species’ diversity (reviewed in [29] ). Individual human genomes can vary by up to 10 % [30], and in maize, only 60 % of genes are found in all the NAM lines [23]. Now that many cultivars of species with complex genomes can be sequenced and analyzed as groups, pan-genome datasets are becoming more available (for example in rice [31, 32] and tomato [33]). These pan-genome sets are valuable for understanding diversity in phenotypes such as disease and drought resistance. However, the issue of effective pan-genome display and interaction by scientific users who may not be command-line savvy has been an ongoing challenge which we have attempted to rectify.

Here, MaizeGDB introduces a pan-genomic approach to hosting a genomic database, leveraging the large number of diverse maize genomes and their associated datasets to quickly and efficiently connect genomes, gene models, expression, methylome, sequence variation, structural variation, transposable elements, and diversity data across genomes so that researchers can easily track the structural and functional differences of a locus and its syntenic orthologs across maize. MaizeGDB provides tools and resources that offer three perspectives to each hosted genome: (1) genomes can be used independently with associated genome-specific data; (2) genomes are associated to the “representative” B73 reference genome and the large sets of accompanying data; and (3) genomes are presented in a pan-genomic framework where gene annotations and sequence variation are interlinked between the various genome assemblies. We believe our cross-genome, pan-genomic framework is unique among databases, but it can be a template for any genomic database poised to host large-scale pan-genomic data.

## Construction and content

The data in MaizeGDB is subdivided into different categories, as described in [16]. Briefly, MaizeGDB is organized based on data or tool type (for example, genomes, SNP diversity, BLAST). Maize genomes are listed on a Genomes page, with links to metadata and downloads; each annotated gene model has its own gene model page. MaizeGDB currently hosts 48 maize genomes in our database, including the 25 NAM founder lines and the most recent version (version 5) of the reference maize genome B73 (Zm-B73-REFERENCE-NAM_5.0, referred to in this article as B73v5). Below we describe how we re-formatted MaizeGDB to become a pan-genomic resource for maize, and how this new format assists users in connecting genomic to functional data.

## Utility and discussion

### Grouping genome assemblies by project

**Fig. 1:**
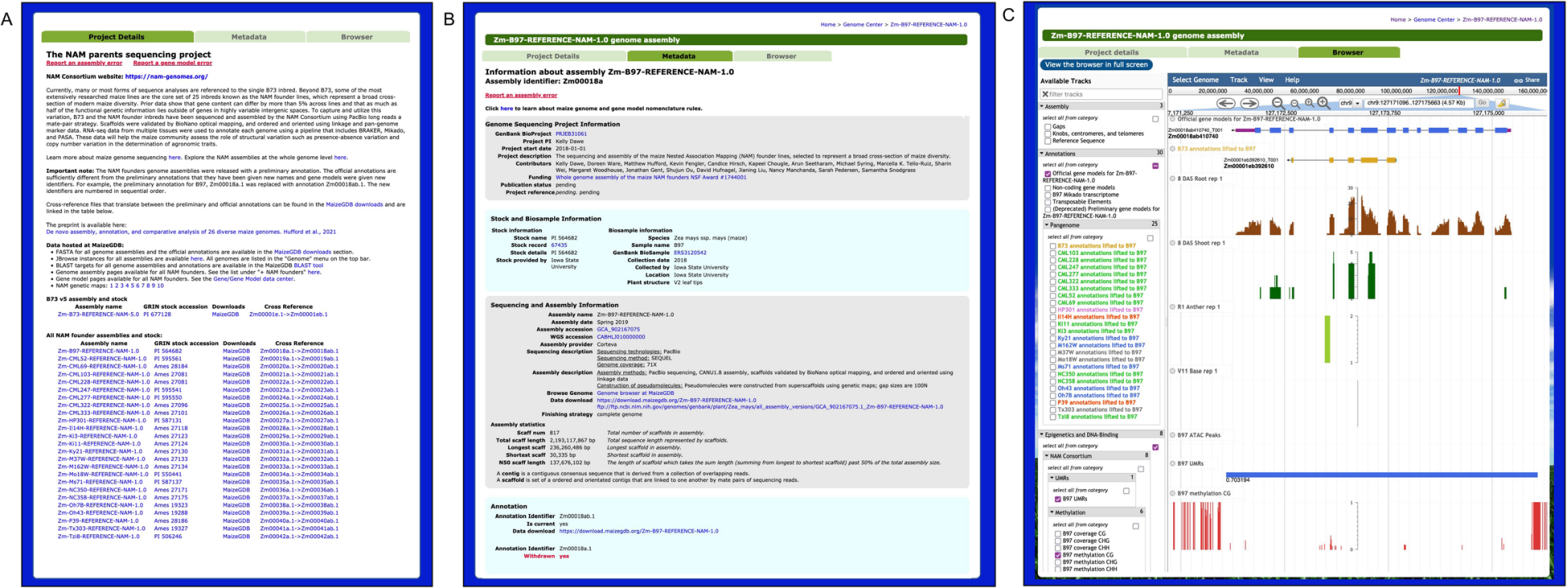
The NAM Genomes pages organized for the NAM founder project (https://maizegdb.org/NAM_project). **A** The “Project Details” page describing the NAM Genome project and listing the genomes associated with the project. **B** The “Metadata” tab for the genome B97 (formally Zm-B97-REFERENCE-NAM-1.0). **B** The “Browser” tab for the genome B97

To facilitate access to the 25 NAM founder genomes, we have reorganized the user interface so that all the NAM founder lines can be found on a single page (https://maizegdb.org/NAM_project), subdivided into three tabs (Fig. 1). The tab “Project Details” (Fig. 1A) lists all the NAM founder genomes together, along with links to their stock accessions and download pages to access data related to these genomes, and contains a description of the NAM project that is germane to all NAM lines. The data on this tab is common to all 25 genomes in this genome set. The tab “Metadata” (Fig. 1B) describes the sequencing, assembly, annotation, and other data specific to a given genome (in this example, B97). The third tab, “Browser”, takes the user to a browser instance of that genome (Fig. 1C).

### The pan-genome JBrowse

One of the challenges of pan-genome visualization is viewing and navigating across multiple genomes, independent of a reference genome. Normally, pan-genome visualization in tools such as IGV or JBrowse is dependent on aligning data from other genomes onto a reference genome, then generating tracks from the reference genomic coordinates of these aligned data. One drawback of this technique is that it is difficult to represent data present in other genomes but absent in the reference genome. However, pan-genomic visualization schemes independent of a reference-genome coordinate system, such as graphical visualization with nodes and edges, can be difficult for users to interpret [34]. One solution we implemented is linking gene models, SNPs, or markers shared between two or more genomes across their JBrowse instances. Until 2020, MaizeGDB presented genome assemblies using GBrowse, a server-side browser software package introduced in 2002 [35]. In 2020, MaizeGDB upgraded to the faster, more modern client-side JBrowse browser software [36]. The NAM founder genomes and B73v5 are now on JBrowse.

**Fig. 2:**
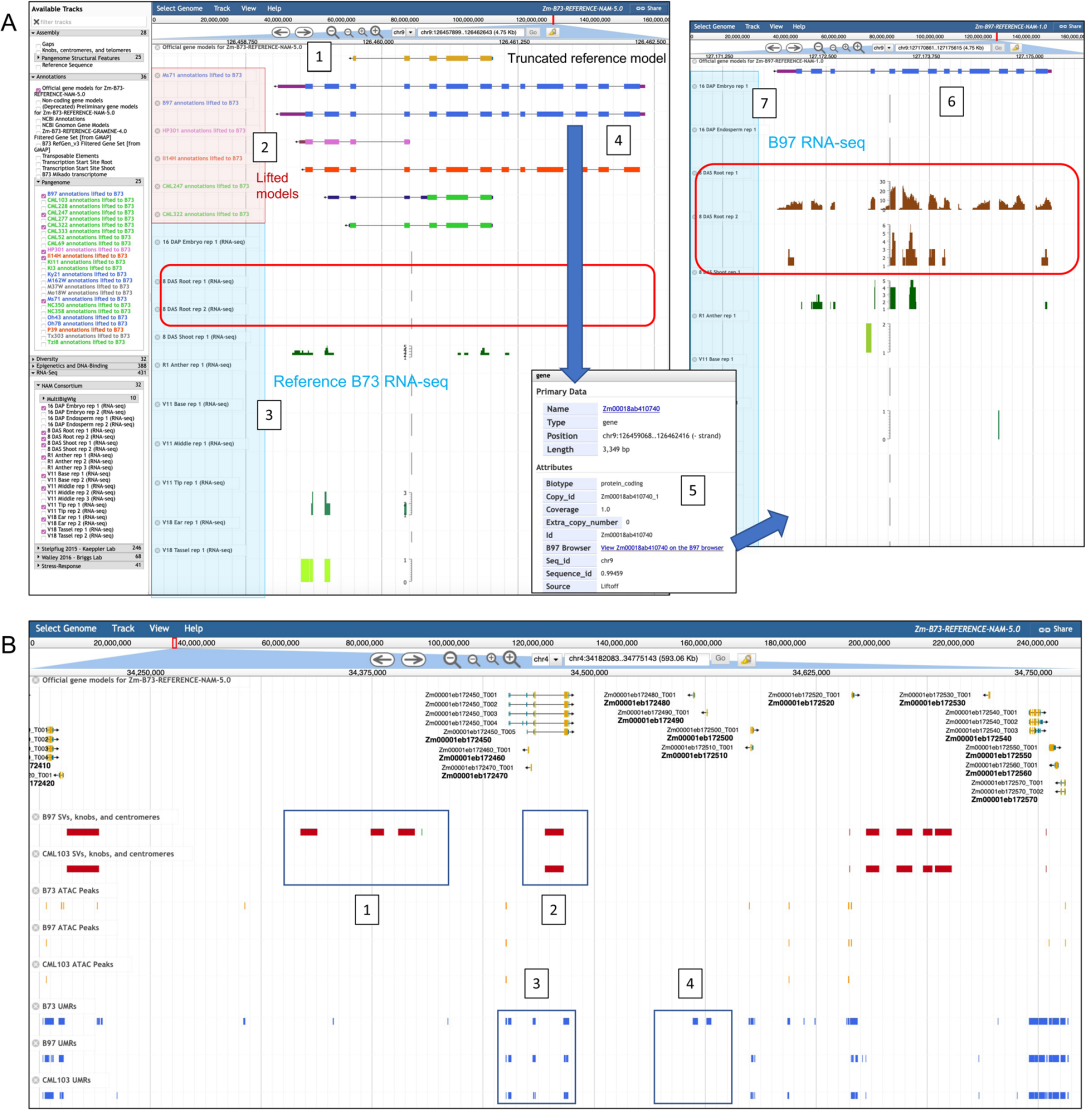
MaizeGDB JBrowse pan-genome optimization. **A** Cross-referencing gene model annotations across the NAM Founders and B73v5. The panel on the left is the B73v5 JBrowse instance, demonstrating that a gene model annotation is truncated in the reference genome (1), but complete in the lifted over tracks (2) for Ms71 and B97, both of which are non-stiff-stalk temperate lines; Il14H, a sweet corn line, has all of the exons present but not the UTRs. HP301 (popcorn), CML247, and CML322 (tropical lines) are also truncated. (3) demonstrates that there is little RNA-seq expression at this locus in B73, and none in root tissue (red box). If a user clicks on the B97 gene model Zm00018ab410740 (4), a pop-up box opens; clicking on the link (5) it will take the user to the location of Zm00018ab410740 in the B97 browser (6). B97 Zm00018ab410740 RNA-seq (7) demonstrates robust expression in root tissue (red box), supporting the B97 gene model annotation, in contrast to the associated B73 locus, which has less RNA-seq support, particularly in root. The Ms71 locus shows similar root expression patterns but Il14H has no root expression (not shown). **B** Large-scale structural variants (SVs) (red = deletions, green = insertions) relative to B73v5 for two lines, B97, and tropical line CML103, and unmethylated regions (UMRs, blue) and ATAC-seq peaks (orange) for B73v5, B97, and CML103. All data are aligned against the reference B73v5 genome. (1) Deletions (red) relative to B73 that are only in B97 (the red spaces represent the regions in B73 missing in B97); these regions are not deleted in CML103, as indicated by the absence of the red spaces in that genome. (2) Deletion shared between B97 and CML103 relative to B73. (3) UMRs present at the same loci in B73, B97, and CML103. (4) UMRs present only in B73

In Fig. 2, we demonstrate our JBrowse cross-genome functionality by linking gene models across the NAM founder lines and B73v5. Using the annotation liftover tool Liftoff [37], we lifted every NAM and B73v5 gene model annotation set to each other (for a total of 676 cross-lifted annotation sets), and generated JBrowse tracks out of the results. Unlike other annotation-lifting tools, Liftoff allows for lifting of gene model annotations across different genome accessions as it takes into consideration structural differences such as inversions between assemblies. In any NAM or B73v5 JBrowse instance, a user can select a track of a lifted over gene model annotation from any other genome to determine if an annotation on the current browser is present in the other genome. If so, the user can click on the lifted gene model annotation feature, which opens a pop-up window containing a link that will take the user to that same gene model located in the JBrowse genome instance corresponding to the lifted annotation track (Fig. 2A). These Liftoff-generated tracks are also useful for identifying regions annotated in other genomes that might have been missed or truncated in the target genome. A region missing an annotation, if coupled with other functional data such as RNA-seq expression and hypomethylation, can suggest that region is likely functional, which is important if the region happens to have a mutational insertion or a SNP that is of interest to the user. The example in Fig. 2 demonstrates an annotation within the non-stiff-stalk temperate line B97 that is truncated in the reference B73v5 genome. Comparing RNA-seq between both genomes demonstrates that there is functional support for the B97 annotation in root, but less functional support for a full annotation at the corresponding B73 locus. Prior to this new cross-browser functionality, a user would have needed to deduce the syntenic relationships between the B73 and B97 gene models, then separately search for these gene models on their respective browser pages in order to compare the differences in expression between these loci.

This cross-genome functionality is also enabled for NAM phenotypic traits which we extracted from [28] and which we have mapped to all the NAM founder lines and B73 (Supplemental Information), and enabled the pan-genome markers which were used to assemble the NAM founder genome superscaffolds into pseudomolecules. In this way, we can link markers and important phenotypic traits across all the NAM genomes and the reference genome. Equally important is that we can identify instances where markers underlying a particular phenotypic trait are missing in a given NAM line.

This cross-browser linkage is a useful way for a researcher to quickly compare the RNA-seq, gene model structure, methylome, transposable element annotation, structural variation, and trait marker information between the loci of two or more genomes to understand the differences in the structure and function of orthologous regions.

### Expanding epigenetic and structural relationships across maize

MaizeGDB had expanded the number of tracks in the reference genome B73v5 JBrowse to include an epigenetic atlas [38] that contains ChIP-seq (Chromatin Immunoprecipitation Sequencing), ATAC-seq (Assay for Transposase-Accessible Chromatin), methyl-seq, as well as DNA-binding sites to further enhance the reference genome’s functional landscape. Epigenetic data can indicate regions of open chromatin and functional gene space, or conversely can identify regions likely to be epigenetically silenced. Such information, along with the RNA-seq data, is crucial to helping researchers determine if a locus of interest is likely to be functional.

MaizeGDB also includes epigenetic data generated by the NAM Consortium [23] including DNA methylation, UMRs (unmethylated regions), and ATAC-seq data mapped to all the NAM founders and B73v5. Additionally, the NAM founder data is mapped onto B73v5 itself, so as to compare differences in the epigenetic landscape across all NAM founders against the reference genome at once (Fig. 2B).

Structural variant (SV) data such as knobs, centromeres, large-scale deletions, and other features for each NAM founder genome generated by the NAM Consortium [23] is displayed as tracks on the NAM founder and B73v5 JBrowse instances. Similar to the epigenetic data, SV data from the other NAM founders have also been projected by the NAM Consortium onto B73v5 (Fig. 2B), enabling researchers to compare differences in large-scale structural variants across all NAM founders against the reference genome.

### Gene models under one pan-gene tab

**Fig. 3:**
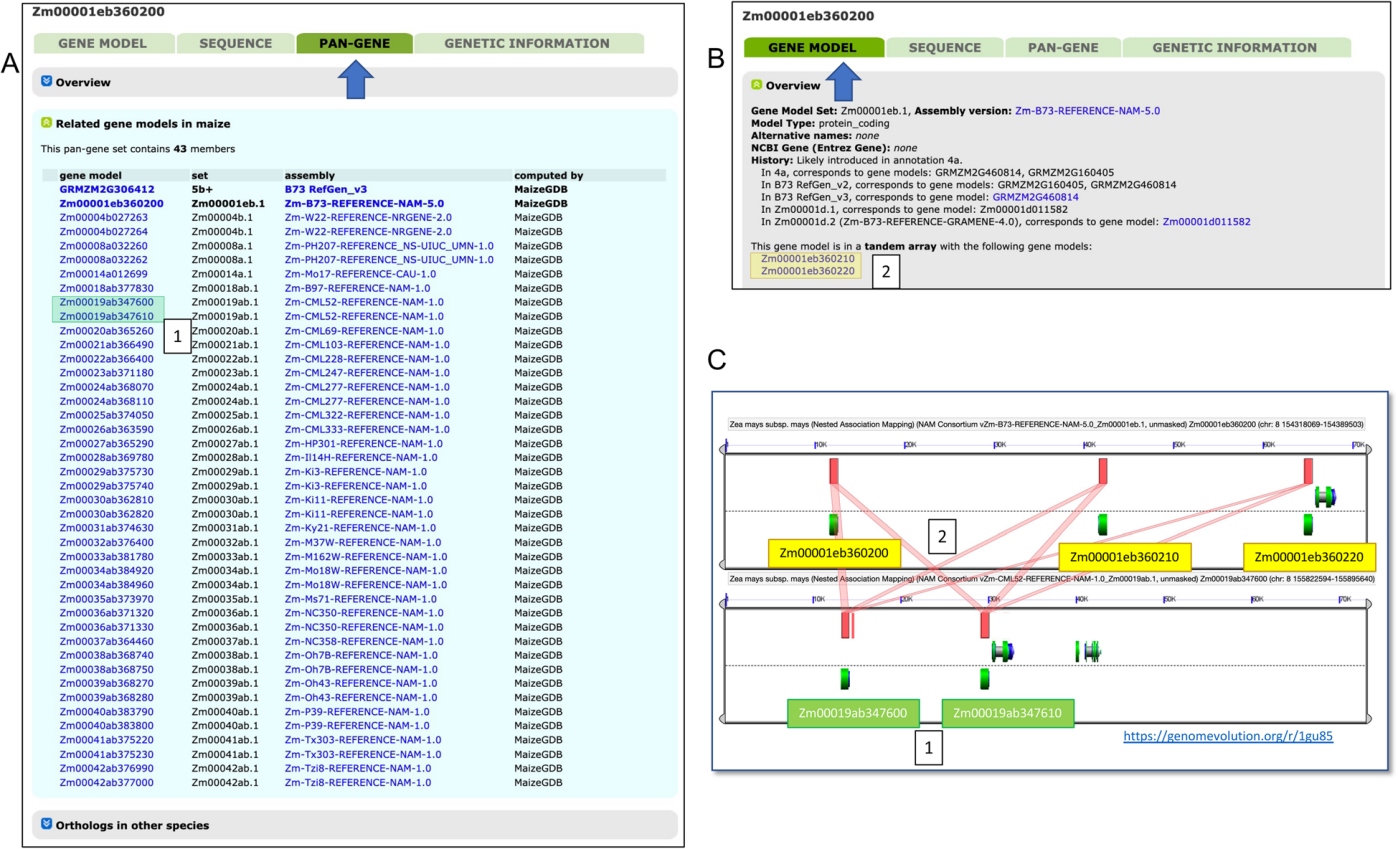
Pan-genome and tandem array information on the gene model pages. Featured is information for the reference B73v5 gene model Zm00001eb360200. **A** The pan-gene tab (arrow) of Zm00001eb360200 and all syntenic orthologs for most maize lines hosted by MaizeGDB. Gene model information for syntenic orthologs (“gene model”) and the name of the genome (“assembly”) is provided. Collapsible sections for “Overview” (top) and “Orthologs in other species” (bottom) provide more information on how the pan-gene relationships were derived, and grass syntenic relationships, respectively. Many genomes have more than one representative; for instance, “Zm-CML52-REFERENCE-NAM-1.0” has two gene models in the set: Zm00019ab347600 and Zm00019ab347610; these are tandem duplications. **B** A partial screen capture of the gene model tab (arrow) with information about gene models that are tandem duplicates of the reference gene model Zm00001eb360200 (2) (Zm00001eb360210 and Zm00001eb360220) for a total of three gene copies, including the reference gene model. **C** A CoGe (https://genomevolution.org/) visualization alignment output of the tandem arrays for B73 and CML52 featured in **A**) and **B**). The green cartoons are gene models; the red boxes are blastn alignments of the coding regions between the two genomes. This image supports the MaizeGDB pipeline determination of tandem array copies in both B73 and CML52

Across all genomes, each gene model in an annotated genome has a page at MaizeGDB where information such as genomic location, genetic map position, gene model structure, transcript and protein sequence, function, expression values, mutational information, and other data (where available) is described (Fig. 3). Before we migrated to the pan-genome approach, this information was subdivided into three tabs: one tab for gene model and expression information, one tab for sequence information of the gene model, and one tab for the genetic information associated with the gene model. With the implementation of the pan-genome approach, we added a new tab that includes pan-gene relationships between a given gene model and syntenic gene models in other maize genomes. Our definition of a pan-gene is a locus in maize that includes all the syntenic orthologs across two or more genomes. The pan-genes for MaizeGDB (Fig. 3 A) were generated by aligning the primary CDS transcripts of all genomes to the primary CDS transcripts of all other genomes with blastn [39], followed by DagChainer [40] on each pairwise blastn output, and then uniting the whole using the Markov Cluster Algorithm (MCL [41], Methods). The pan-gene tab shows all members of the resulting pan-gene set associated with the gene model of interest.

We also identified tandem gene arrays via self CDS transcript blastn hits within a 300 kb window (Methods), and report all the other blastn hits that fall within that tandem array besides the represented gene in the gene model page (Fig. 3B C). This provides information on both a gene model’s retention across maize, and its local copy number. Together, these features on the new pan-gene tab allow users to understand the copy number and syntenic relationships of any given gene model to all other gene models in maize.

### Compare chromosome-level structural variation across maize genomes with CViTjs

**Fig. 4:**
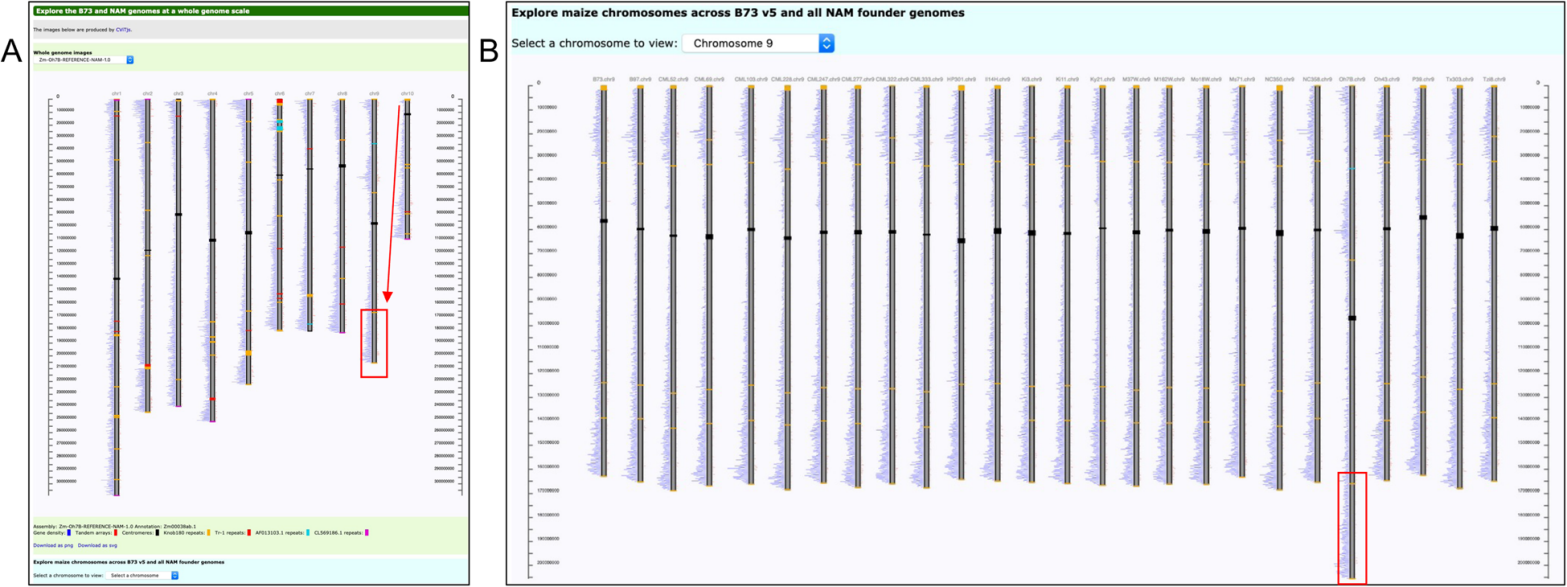
CViTjs viewer. **A** Whole-genome view of NAM founder Oh7B. Chromosome 5 is an example of typical gene density, with the highest density near the ends of each arm. Chromosome 9 is a clear exception with peak gene densities at the end and midway down the proximal arm, whereas Chromosome 10 lacks a clear gene density peak on its proximal arm. The translocation from the proximal arm of Chromosome 10 (arrow) to the distal arm of Chromosome 9 (rectangle) is highlighted. **B** Display of chromosome 9 across all 26 genomes. Here it is evident that Chromosome 9 is distinctly larger in Oh7B compared Chromosome 9 in the other NAM founders; the rectangle highlights the region of Oh7B Chromosome 10 translocated to Chromosome 9

Macro views of genomic features at the whole-genome level can reveal patterns undetectable at close range. MaizeGDB makes use of the tool CViTjs (Chromosome Visualization Tool-javascript; https://github.com/LegumeFederation/cvitjs) to generate whole-genome views of B73v5 and the NAM founder assemblies. CViTjs is a flexible Javascript application that displays features, categorized as points or ranges, displayed as rectangles, circles or histograms, using a specified color or heat color range on pseudomolecules, linkage groups, or chromosomes. Any type of feature can have an attached label. CViTjs can be used to display genetic maps, genomic features, or cytological features. The CViTjs images layer gene and tandem repeat densities along with centromere and knob regions on whole genome views for B73v5 and the NAM founders. Alternative views show the same features on each of the 10 chromosomes across all 26 genomes. Figure 4 shows an example for the NAM founder line Oh7B which has a translocation from Chromosome 10 to Chromosome 9 that is elegantly displayed in CViTjs.

### RNA-seq visualization via qTeller and the NAM founders

qTeller is a program to visualize RNA expression in a given gene, a genomic coordinate, or pair of genes across multiple RNA-seq datasets (manuscript submitted). It allows a user to visually compare gene expression of a selected gene model in different tissues, time points, and conditions, or to compare gene expression between two gene models. It also accepts a list of gene models and outputs a file with each gene model’s expression abundances for each tissue/condition library the user selected.

**Fig. 5:**
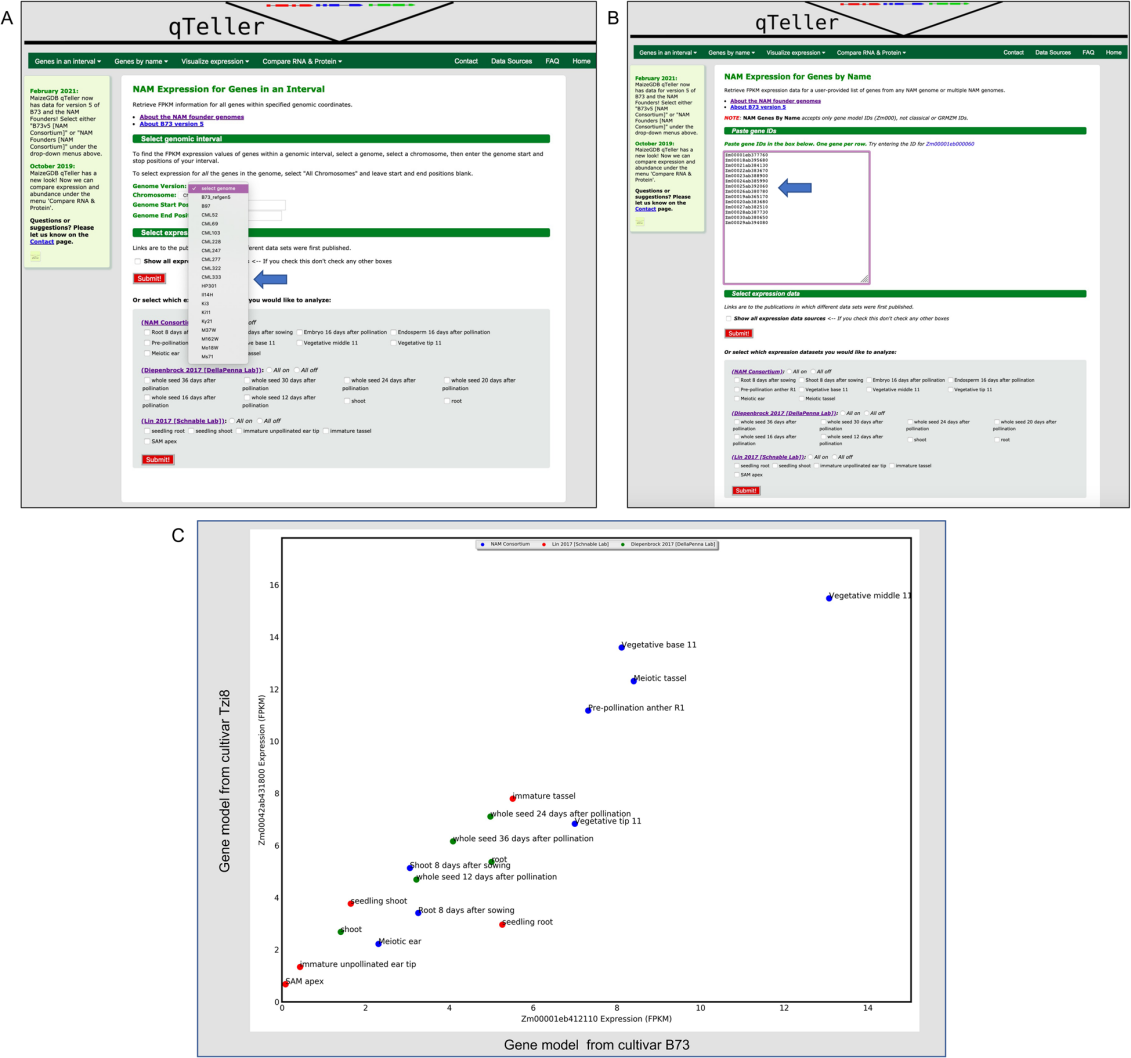
MaizeGDB NAM Multi-Genome qTeller instance. **A** The “Genes in an Interval” feature, with a drop-down menu (arrow) to select the NAM genome of interest. **B** The “Genes by Name” feature, where gene models from several different genomes (distinguished by the different prefixes: Zm00001eb = B73v5, Zm00018ab = B97, etc.; see Nomenclature section below) can be entered to retrieve expression results across genomes. **C** Comparison of two syntenic gene models from two different genomes: Zm00001eb412110 (B73) and Zm00042ab431800 (Tzi8). The X and Y coordinates represent the respective FPKM values for each gene model. Three different RNA-seq expression datasets are represented (blue, green, red). The expression values between the two gene models are fairly consistent, which is often true of syntenic loci

MaizeGDB has hosted qTeller since 2018 (https://qteller.maizegdb.org/), and has updated the tool to include a method of comparing protein abundances and studying multiple genomes. Initially, only genes from version 4 of the reference maize genome B73 were represented. Because the NAM founder sequencing project also sequenced RNA-seq data for ten tissues across all the NAM founder lines, we were able to make a NAM founder pan-genome qTeller instance where a user could compare the RNA expression of a gene in one NAM genome to the RNA expression of a gene in another NAM genome. The genomes and the RNA-seq data were all generated exactly the same way by the same sequencing group, allowing a more equal comparison across genomic datasets than would be expected of RNA-seq data between genomes generated by different labs and under different conditions. This qTeller instance thus permits a user to easily compare normalized expression profiles across shared genes within maize (Fig. 5).

### Genome nomenclature for multiple genomes

MaizeGDB serves as the clearinghouse for maize nomenclature in collaboration with the Maize Nomenclature Committee, including for genome assemblies and annotations. With multiple genomes hosted at MaizeGDB and especially the set of NAM founder assemblies that require names identifying them as being members of a set, it was necessary to establish consistent naming conventions for the genome assemblies, annotation sets, and gene models that are both human and machine readable. Doing so also assists computational analyses of all NAM assemblies and annotations. The guidelines can be found here https://documents.maizegdb.org/nomenclature/maize_assembly_nomenclature_2016_update.pdf.

All NAM founder assemblies are named with the pattern: Zm-[cultivar]-REFERENCE-NAM-1.0, for example, Zm-B97-REFERENCE-NAM-1.0. A minor exception is the B73 assembly, which as the 5th version of the representative maize genome, is named Zm-B73-REFERENCE-NAM-5.0.

All NAM assemblies are also assigned numbered identifiers of the form Zm[ddddd][l]. For the NAM assemblies these are Zm00001e (B73v5), and Zm00018a (B97v1) through Zm00042a (Tzi8v1). These identifiers are used as prefixes for the gene model names, with the addition of one more letter indicating the annotation version. As preliminary annotations were released before the official annotations, the official annotations use prefixes Zm00018ab - Zm00042ab, where ‘b’ indicates the second annotation. Gene model numbers are numbered sequentially across the chromosomes, separated by 10. For example: Zm000018ab000100, Zm000018ab000110, Zm000018ab000120, et cetera. Chromosome numbers are not encoded in the names.

## Conclusions

Increased efficiency and quality of genome sequencing and assembly, and its exponentially lower cost have accelerated the pace and number of genome assemblies being released. Genomic databases will not only host many more genomes, but also provide resources to integrate and compare across different genomes. MaizeGDB has developed multiple means for harnessing the growing number of maize genomes to explore the diversity and complexity of maize. Using the high-quality NAM founder genomes as a gold standard, we compiled pan-gene sets accessible through each gene model page, made jumping between genome browsers possible, implemented cross-genome structural variant comparisons, and expanded RNA-seq analysis to be tractable across multiple genomes. These recent updates at MaizeGDB can serve as a template for other databases to manage large-scale pan-genomes of any species.

## Methods

Lifted Annotations: Annotations were lifted across genomes using the tool Liftoff [37] using default parameters.

MaizeGDB Pan-Genome: Scripts for the pan-genome and tandem duplicate relationships can be found in the MaizeGDB GitHub repository https://github.com/Maize-Genetics-and-Genomics-Database/Pan-Genome. The MaizeGDB pan-genome was generated using a pipeline based on Steven Cannon’s pan-genome pipeline structure developed at Soybase [42] and Legume Information System [43]), with a combination of blastn [39], DagChainer [40], and MCL [41]. The CDS genomic fasta files for the primary or canonical transcripts of each genome were selected, and from these, blast databases were generated. Pairwise blastn alignments (including self alignments) were run with the parameters blastn -query ${q} -db ${s%.*} -perc_identity 95 -evalue 1e-10 -outfmt “6 std qlen slen qcovs”. Outputs were merged with the genomic coordinates of each canonical transcript and formatted for DagChainer. DagChainer parameters were perl DAGCHAINER/run_DAG_chainer.pl -i ${sample} -D 1,000,000 -g 40,000 -A 5. MCL parameters were mcl -I 1.2 -te 20 --abc -o.

Tandem duplicate relationships: Self-self blastn alignments from the above were filtered so that non-self gene models falling within a 300 kb window were selected. These were then run through MCL using the command mcl I 2.0 -te 20 --abc -o.

## Supplementary Information

**Additional file 1: Supplemental Figure 1.** SNP data for the NAM founders mapped onto the reference B73 genome. Represented on the browser are a subset of SNPs from non-stiff-stalk NAM lines (blue), the popcorn line HP301 (pink), the sweet corn lines (orange), and the tropical lines. SNPs are color-coded based on trait. 1) SNPs missing from the sweet corn lines IL14h and P39. 2) By clicking on an adjacent SNP in Il14h, a pop-up box opens, where a link 3) will take the user to the SNP on the IL14h browser. This experiment can be replicated at the following link https://jbrowse.maizegdb.org/?data=IL14H&loc=chr2%3A209060001..210188000&highlight=chr2%3A209624165..209624272&tracks=gwas_snps

## Data Availability

All data described in this work can be found at https://maizegdb.org/. Software described can be found in https://github.com/Maize-Genetics-and-Genomics-Database and https://github.com/LegumeFederation/cvitjs.

## Abbreviations

MaizeDB: Maize Database
MaizeGDB: Maize Genetics and Genomics Database
NAM: Nested Associated Mapping
MCL: Markov Cluster Algorithm
ChIP-seq: Chromatin Immunoprecipitation Sequencing
ATAC-seq: Assay for Transposase-Accessible Chromatin
SV: Structural variant
CViTjs: Chromosome Visualization Tool-javascript
