# Supplementary material for: A pan-genomic approach to genome databases using maize as a model system
Source: BMC Plant Biol. 2021 Aug 20;21:385. doi: 10.1186/s12870-021-03173-5 (PMC8377966; doi:10.1186/s12870-021-03173-5)
Supplement: Supplementary file 1 — Additional file 1: Supplemental Figure 1. SNP data for the NAM founders mapped onto the reference B73 genome. Represented on the browser are a subset of SNPs from non-stiff-stalk NAM lines (blue), the popcorn line HP301 (pink), the sweet corn lines (orange), and the tropical lines. SNPs are color-coded based on trait. 1) SNPs missing from the sweet corn lines IL14h and P39. 2) By clicking on an adjacent SNP in Il14h, a pop-up box opens, where a link 3) will take the user to the SNP on the IL14h browser. This experiment can be replicated at the following link https://jbrowse.maizegdb.org/?data=IL14H&loc=chr2%3A209060001..210188000&highlight=chr2%3A209624165..209624272&tracks=gwas_snps [file 12870_2021_3173_MOESM1_ESM.pdf]

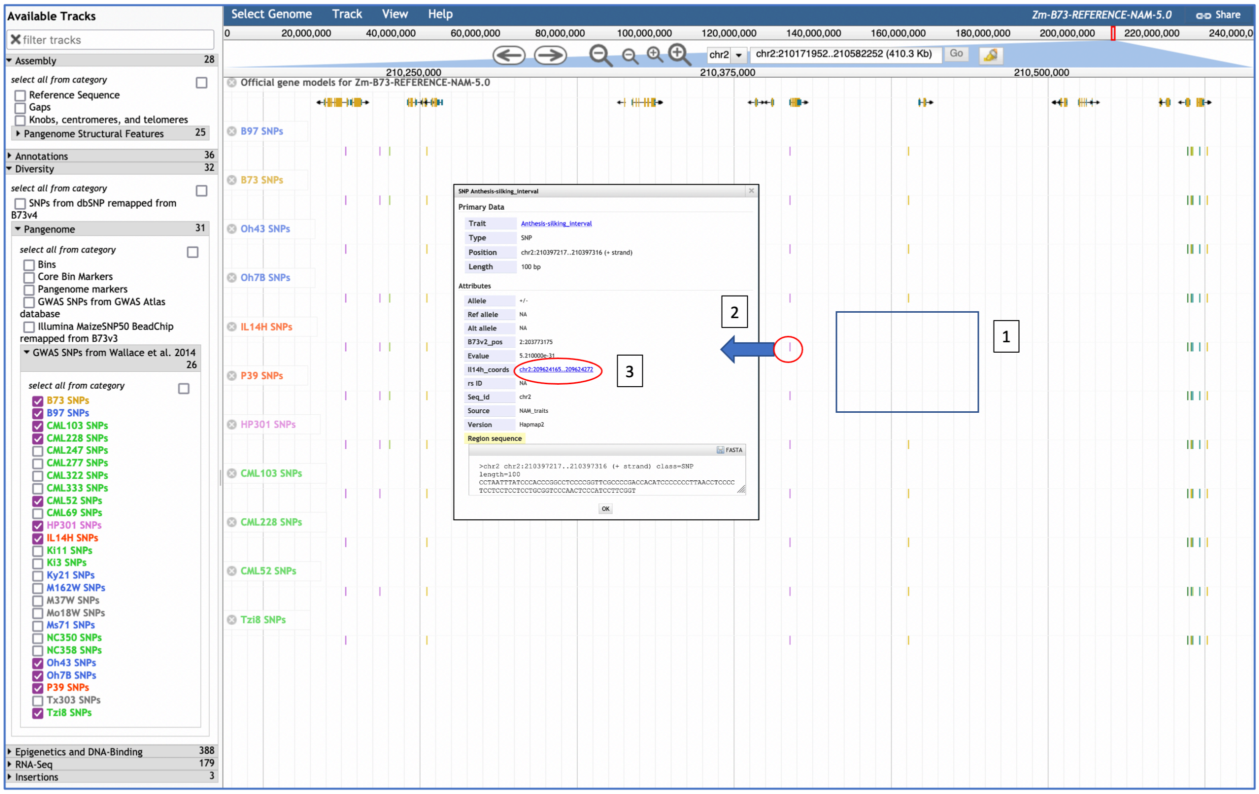

Supplemental Figure 1: SNP data for the NAM founders mapped onto the reference B73 genome. Represented on the browser are a subset of SNPs from non-stiff-stalk NAM lines (blue), the popcorn line HP301 (pink), the sweet corn lines (orange), and the tropical lines. SNPs are color-coded based on trait. 1) SNPs missing from the sweet corn lines IL14h and P39. 2) By clicking on an adjacent SNP in Il14h, a pop-up box opens, where a link 3) will take the user to the SNP on the IL14h browser. This experiment can be replicated at the following link https://jbrowse.maizegdb.org/?data=IL14H&loc=chr2%3A209060001..210188000&highlight=chr2%3A209624165..209624272&tracks=gwas_snps
